# Maternal and neonatal service usage and determinants in fragile and conflict-affected situations: a systematic review of Asia and the Middle-East

**DOI:** 10.1186/s12905-017-0379-x

**Published:** 2017-03-15

**Authors:** Saji S. Gopalan, Ashis Das, Natasha Howard

**Affiliations:** 1 London School of Hygiene and Tropical Medicine (LSHTM), London, UK; 2 The World Bank, Washington, DC USA

**Keywords:** Fragile and conflict-affected, Maternal and neonatal health, Care-seeking, Service usage, Asia, Middle-East

## Abstract

**Background:**

Fragile and conflict-affected situations (FCS) in Asia and the Middle-East contribute significantly to global maternal and neonatal deaths. This systematic review explored maternal and neonatal health (MNH) services usage and determinants in FCS in Asia and the Middle-East to inform policy on health service provision in these challenging settings.

**Methods:**

This systematic review was conducted using a standardised protocol. Pubmed, Embase, Web of Science, and selected development agency websites were searched for studies meeting inclusion criteria. Studies were assessed for methodological quality using an adapted evaluation tool. Qualitative and quantitative data were synthesized and pooled odds ratios generated for meta-analysis of service-usage determinants.

**Results:**

Of 18 eligible peer-reviewed studies, eight were from Nepal, four from Afghanistan, and two each from Iraq, Yemen, and the Palestinian Territories. Fragile situations provide limited evidence on emergency obstetric care, postnatal care, and newborn services. Usage of MNH services was low in all FCS, irrespective of economic growth level. Demand-side determinants of service-usage were transportation, female education, autonomy, health awareness, and ability-to-pay. Supply-side determinants included service availability and quality, existence of community health-workers, costs, and informal payments in health facilities. Evidence is particularly sparse on MNH in acute crises, and remains limited in fragile situations generally.

**Conclusions:**

Findings emphasize that poor MNH status in FCS is a leading contributor to the burden of maternal and neonatal ill-health in Asia and the Middle-East. Essential services for skilled birth attendance and emergency obstetric, newborn, and postnatal care require improvement in FCS. FCS require additional resources and policy attention to address the barriers to appropriate MNH care. Authors discuss the ‘targeted policy approach for vulnerable groups’ as a means of addressing MNH service usage inequities.

## Background

The United Nations’ post-2015 agenda calls for specific attention to address maternal and neonatal health (MNH) in fragile and conflict-affected situations (FCS). Evidence indicates that without improving MNH service quality and usage in FCS, maternal and child health indicators will not improve sufficiently to achieve Sustainable Development Goal targets [1]. To implement strategic MNH interventions, FCS lack basic information on usage of MNH services and factors affecting access to MNH care.

Globally, 1.2 billion people inhabit fragile countries, while 800 million live in situations of conflict [1]. FCS are defined variously by different agencies [1–3]. However, key criteria include government not delivering core social services, instable governance with weak institutionalization and accountability, low socioeconomic and human-development indicators, and threats to security and right to life from routine internal and external conflicts. Fragility and conflict coexist in many countries (e.g. Afghanistan, Palestinian Territories) [2], while post-conflict categorisations (e.g. Nepal), can still include fragility due to weak governance and social services delivery [1–3]. Weak governance, violent political conflict and adverse living situations challenge necessary improvement of health and living standards in FCS, and gradually impede global economic momentum [3, 4].

Appropriate and equitable MNH service usage is prerequisite to reducing maternal and newborn ill-health, especially in FCS [5]. Evidence indicates that providing skilled birth attendance (SBA) and addressing complications during pregnancy is potential to reduce 13–33% of maternal deaths [6]. Similarly, up to two-thirds of newborn deaths are preventable with provision of essential newborn care at birth and during the first week of life [6]. FCS perform poorly on MNH as they lack the governance and infrastructural capacity to provide SBA, emergency obstetric and newborn care (EmONC), contraception, and address related maternal and neonatal complications [5].

Although poor MNH status is a concern in FCS globally, FCS in Asia and the Middle-East are particularly relevant, with poor MNH indicators relegating several low- and middle-income FCS as some of the worst globally, e.g. Afghanistan and Yemen [7]. Several Asian countries did not achieve MDGs 4 and 5, due to fragility and conflict (e.g. Nepal, Afghanistan) [7–10]. The average maternal mortality ratio in this region is 200 deaths per 100,000 live births [9, 10]. Long-term fragility is one key reason. For instance, Yemen, a chronically fragile country, has a higher maternal mortality ratio of 500/100,000 [9]. Among other top contributors of maternal deaths in this region, the Palestinian Territories and Iraq are prone to conflicts, whereas Nepal has been fragile for years [2]. Asian FCS have some of the highest rates of neonatal mortality globally. For example, Afghanistan, a conflict-affected country, accounts for 2% (i.e. 36,777 deaths) of global neonatal mortality [11–13].

FCS in Asia and the Middle-East require substantial informing evidence on MNH service usage and its determinants to help streamline policy strategizing, yet available information is limited [1]. As levels of fragility and conflict vary among FCS, so does their ability to deliver MNH services [7]. Further MNH investments in the FCS of this region are unlikely to be cost-effective without a greater understanding of MNH service usage and identification of demand-and supply-side challenges and opportunities.

### Objectives

The aim of this systematic review was to identify and analyse the literature on maternal and neonatal service-usage and potential determinants in FCS of Asia and the Middle-East. This region was selected because FCS here are leading contributors to the global maternal and neonatal morbidity burden and contribute relatively more eligible literature enabling a systematic review. Objectives were to (i) summarise the main findings on maternal and neonatal service-usage and (ii) identify key determinants of maternal and neonatal service usage. Implications for policy and practice were considered.

## Methods

### Protocol and registration

Using the criteria shown in Table 1, eight low- and middle-income FCS were selected (i.e. Afghanistan, Iraq, Myanmar, Nepal, the Palestinian Territories, Syria, Timor-Leste, Yemen). As FCS were defined through national scoring, no attempt was made to include additional countries with sub-national areas of fragility (e.g. India) that did not fulfil scoring criteria. A pre-designed protocol guided the review processes, including search strategy, study selection, data extraction, quality assessment, and data synthesis. Reporting uses PRISMA (Preferred Reporting Items for Systematic Reviews and Meta-analyses) guidelines [13]. This review is registered with PROSPERO (International Prospective Register of Systematic Reviews) at the National Institute of Health Research, USA, as CRD42014014537 [14].

**Table 1:** Conceptual definitions

Concept	Definition
Asia	The largest continent in the world, occupying the eastern part of the Eurasian landmass and its adjacent islands, and bordered by the Ural Mountains, Arctic, Pacific and Indian Oceans, and Mediterranean and Red Seas. Countries eligible for inclusion are those territorially part of the Asian continent without any inter-continental territorial disputes [48].
Middle East	A term referring, generally, to the geographical area and countries between the Black Sea to the north and the Arabian Sea to the south, including Iran and Egypt (e.g. Egypt, Iraq, Iran, Israel/Palestine, Jordan, Kuwait, Lebanon, Oman, Qatar, Saudi Arabia, Syria, Turkey, United Arab Emirates, Yemen) [48].
Fragile and conflict-affected situation (FCS	The World Bank Harmonised List of Fragile Situations (FY14) was used, as it is accepted by several development banks and agencies [49]. Criteria are either (a) a harmonized average country performance Indicator assessment (CPIA) rating of 3.2 or less, or (b) the presence of a UN and/or regional peace-keeping or peace-building mission during the past three years. CPIA consists of performance rating against 16 criteria in four clusters: (i) economic management, (ii) structural policies, (iii) policies for social inclusion/equity, and (iv) public-sector management and institutions [50].
Health services usage	Use of health services and supplies, it is commonly measured in terms of patterns or rates per unit of population at risk during a specified time-period [49, 51].
Maternal services	Any preventive and curative services related to pregnancy, childbirth and the postpartum period [51]. Services include antenatal care, any delivery-related care (e.g. home delivery, institutional delivery, skilled attendance), emergency obstetric care, and postpartum care [49, 51].
Newborn services	Any preventive and curative postnatal services during the first 28 days after birth, including early breastfeeding within an hour of birth, exclusive breastfeeding, treating illnesses and symptoms, and newborn vaccination [49, 51].

### Information sources and search

The literature search was conducted in April 2014. First, authors searched electronic databases Embase, PubMed, and Web of Science systematically. Second, authors purposefully searched agency websites of Asian Development Bank, DFID, OECD, World Bank, and WHO. Third, authors identified potentially relevant citations from included studies and hand-searched them in relevant journals identified. Search strategies and terms were adapted as appropriate for each database and site, with a combination of MeSH and non-MeSH terms using Boolean operators “AND” and “OR”. Key search topics were:

*“maternal health/care” [MeSH] OR “childbirth” [MeSH] OR “institutional delivery” OR “skilled birth attendance” OR “antenatal /prenatal” OR “postnatal/postpartum ” OR “neonatal/perinatal/newborn” OR “infant” OR “child care/health” or “under-five care/health” AND “fragile setting/context” OR “crisis setting” OR “conflict-affected region/areas” OR “Afghanistan” OR “Nepal” OR “Myanmar/Burma” Or “Timor-Leste/East Timor” OR “Yemen” OR “West Bank and Gaza/Palestine/Palestinian Territories” OR “Iraq” OR “Syria” OR “ Asia” OR “ Middle-East”*

### Eligibility criteria and study selection

Inclusion and exclusion criteria were established through an iterative process. Authors agreed initial selection criteria based on the research question (i.e. “*What are the status and determinants of maternal and neonatal health and service-usage in FCS of Asia and Middle-East*?”), focusing on primary research. Any primary study (i.e. peer-reviewed article reporting quantitative, qualitative and mixed-methods design) was considered if published in English during 2000–2013 and reporting maternal or neonatal service usage or determinants in Asian and Middle-Eastern FCS.

Policy evaluations, reviews and discussion papers were excluded. Studies given a low quality-score during methodological assessment were also excluded. The first author reviewed title, abstract and key words of all articles retrieved. Those articles meeting eligibility criteria were selected for full review. A second author checked selection of relevant abstracts and full articles. Any disagreements prompted another round of scrutiny until consensus was reached.

### Data collection

Data were extracted from each record into an Excel spreadsheet. Headings included: lead author, co-authors, year of publication, type of source (e.g. PubMed, website), study setting, study design, outcomes of interest, number and type of participants, participant selection criteria, sample size, data analysis and reporting, limitations, and methodological quality score.

### Data items

Study outcomes were restricted to service-usage for any of antenatal care (ANC), institutional delivery, skilled birth attendance (SBA), emergency obstetric and neonatal care (EmONC), postnatal care (PNC), early breastfeeding, newborn consultation for any perceived morbidity, and newborn vaccination. Any ANC was considered (e.g. any visit, all four visits, ANC from a skilled or unskilled provider). For PNC, any visit and PNC from a skilled or unskilled provider were considered. Factors affecting maternal and neonatal health services usages were included in the synthesis.

### Risk of bias in studies

Authors adapted the McGill University Mixed-Methods Appraisal Tool (MMAT) [15], which has been used to assess methodological quality of both mixed-methods and primary cross-sectional studies (Table 2). In adapting this tool, authors excluded irrelevant indicators (e.g. are measurements appropriate?) and included new ones (e.g. was there any recall bias in reporting data?). Nine indicators were considered for both quantitative and qualitative studies, with each scored 1 if present and zero otherwise. Summative scores indicated overall study quality (e.g. six and above was considered high quality, 4–5 medium quality, less than four low quality and therefore excluded). For mixed-method studies, the maximum summative score was 12 (i.e. nine and above was high quality, 6–8 was medium quality, less than 6 was low-quality and therefore excluded). Two authors assessed quality independently, with disagreements resolved by discussion.

**Table 2:** Methodological quality assessment criteria adapted from MMAT

Assessment criteria	Indicators
Scientific rigor of data collection, analysis and reporting of qualitative studies	Are the sources of qualitative data (i.e. informants, observations, documents) relevant to address the research question?
	Is the process for analysing qualitative data relevant to address the research question?
	Is appropriate consideration given to how findings relate to the context (e.g. setting in which data were collected?
	Is appropriate consideration given to how findings relate to researchers’ influence (e.g. through their interactions with participants)?
Scientific rigour of data collection, analysis and reporting of quantitative studies	Is the sampling strategy relevant to address the quantitative research question (quantitative aspect of the mixed methods question)?
	Is the sample representative of the population under study?
	Is there an acceptable response rate (e.g. 60% or above)?
	Are the statistical methods used appropriate for measurement?
	Was there any recall bias in reporting data?

### Summary measures and data synthesis

Summary measures were percentages and odds ratios (ORs) with a confidence interval of 90, 95 or 99% significance. Based on the heterogeneity of data, a narrative synthesis was conducted, combining both qualitative and quantitative data. If feasible, quantitative data were subjected to meta-analysis using fixed effects with odds ratios pooled for similar determinants in particular services. Forest plots were generated for each determinant with odds ratios and confidence intervals displayed. Heterogeneity among studies was tested using I [1] statistics in Stata 13. Narrative synthesis was used for qualitative data on determinants of MNH service usage to describe and compare findings across settings, types of services and population groups.

## Results

### Study selection

**Fig. 1:**
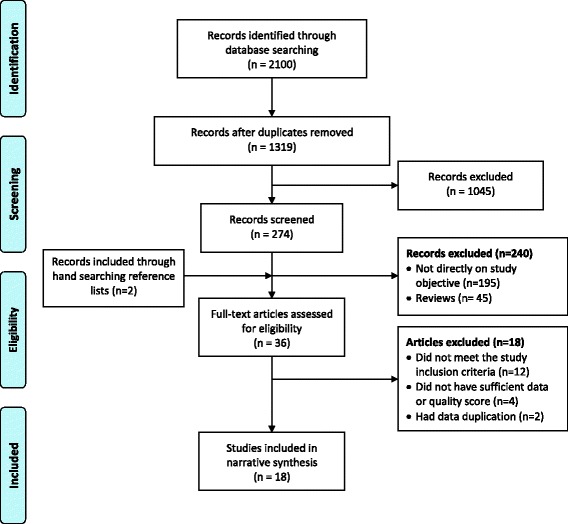
Flow chart of study selection

Figure 1 details the stages of study selection. Initially, after removing duplicates 1319 records were identified as potentially relevant. After excluding irrelevant articles, 274 abstracts were screened, of which 36 were eligible for full text review. Finally, 18 peer-reviewed articles were selected for inclusion.

### Study characteristics and methodological quality

Table 3 shows that among 18 studies included, eight (44%) were from Nepal [16–23], four (22%) from Afghanistan [24–27], and two (11%) each from Iraq, Yemen, and the Palestinian Territories [28–33]. Fourteen (77%) were quantitative studies (i.e. 11 cross-sectional surveys, 2 (11%) used national health survey data, 1 prospective cohort study), two (11%) were qualitative, and two (11%) used mixed-methods designs. All studies included met the required methodological quality criteria suggested by MMAT (Table 2) particularly for data collection, analysis, and reporting. Twelve (66%) studies received a high methodological quality score and 7 (38%) received a medium score. Studies exhibited a few minor quality issues as described below.

**Table 3:** Study characteristics and quality score, ordered by country and year

Author (year)	Country	Design	Sample	Score
Newbrander et al. (2013) [26]	Afghanistan	Qualitative	30 IDIs and 29 FGDs with community members in 5 districts	6/9
Rahmani and Brekke (2013) [27]	Afghanistan	Qualitative	12 IDIs with pregnant/recently-delivered women and 15 IDIs with providers in 2 provinces	6/9
Hirose et al. (2011) [24]	Afghanistan	Cross-sectional	411 paired couples surveyed at 1 regional hospital	7/9
Mayhew et al. (2008) [20]	Afghanistan	Cross-sectional	9917 recently-delivered (2 years) women surveyed in 33 provinces	5/9
Khorrami et al. (2008) [25]	Afghanistan	Cross-sectional	292 women inpatients with obstetric complaints surveyed at 1 hospital	5/9
Najem and Al-Deen (2011) [28]	Iraq	Cross-sectional	251 primipara postnatal mothers surveyed at 1 hospital	4/9
Siziya et al. (2009) [29]	Iraq	Secondary survey	22,980 recently-delivered (1 year) MICS participants	8/9
Sharma et al. (2014) [21]	Nepal	Cross-sectional	240 recently-delivered (1 year) women surveyed in 1 district.	5/9
Choulagai et al. (2013) [16]	Nepal	Cross-sectional	2481 recently-delivered (1 year) women surveyed in 3 districts	7/9
Karkee et al. (2013) [19]	Nepal	Prospective cohort	700 pregnant women in 1 district	7/9
Shrestha et al. (2012) [22]	Nepal	Cross-sectional	732 married reproductive-age women	6/9
Ulak et al. (2012) [23]	Nepal	Cross-sectional	352 mothers of infants attending vaccination	6/9
Devkota and Bhatta (2011) [17]	Nepal	Cross-sectional	71 mothers of newborns	4/9
Dhakal et al. (2011) [18]	Nepal	Cross-sectional	150 recently-delivered women	7/9
Dhaher et al. (2008) [32]	Palestine	Cross-sectional	264 postpartum outpatient women	7/9
Giacaman et al. (2007) [33]	Palestine	DHS survey	2158 women residing in the West Bank and Gaza Strip	7/9
Kempe et al. (2013) [31]	Yemen	Mixed-methods	220 women with childbirth experience in urban/rural areas	7/12
Basaleem (2012) [30]	Yemen	Mixed-methods	1678 women surveyed and 11 FGDs with men and women	9/12

None of the quantitative studies was nationally representative or generalisable. Data analysis was not presented with considerable details in Devkota and Bhatta [17], Khorrami et al. [25], Mayhew et al. [20] or Najem and Al-deen [28]. Khorrami et al. [25], Mayhew et al. [20], Najem and Al-deen [28], Sharma et al. [21] and Ulak et al. [23] did not provide sufficient details on study settings. Three studies (e.g. Devkota and Bhatta [17], Karkee et al. [19], Shrestha et al. [22]) focused on remote fragile settings. Dhaher et al. [32], by examining factors affecting postnatal care in Palestine, reflected the status of postpartum women in a conflict-affected setting. Among qualitative studies, Rahmani and Brekke [27] did not triangulate information from several data sources.

Neither mixed-methods study (i.e. Kempe et al. [31], Basaleem et al. [30]) was nationally representative and generalisability was not addressed. Kempe et al. [31], did not give adequate description on the processes of data analysis or the necessity of using qualitative data. Basaleem et al. [30] used a mixed-methods design to explore maternal health and influence of women’s autonomy in Yemen. In this study, details on study setting and quantitative data analysis were not clearly explained, while statistical analyses of determinants were not in-depth [31].

### Service-usage

Table 4 provides service-usage findings. Three studies (17%) described ANC usage [16, 17, 30]. Overall, at least one ANC visit was above 50% in both Nepal and Yemen [16, 17, 30]. More than 60% of Yemeni women obtained ANC from a professionally trained provider in the first trimester [16].

**Table 4:** Maternal and neonatal service usage, ordered by service type and % usage

Service	Study	Outcome	% usage
Antenatal care (ANC)	Basaleem (2012) [30]	Professional ANC	97.7%
		ANC in 1^st^ trimester	60%
	Choulagai et al. (2013) [16]	4+ ANC	57%
	Devkota and Bhatta (2011) [17]	At least one ANC	71.8%
Skilled birth attendance (SBA)	Basaleem (2012) [30]	SBA usage	50%
	Choulagai et al. (2013) [16]	SBA usage	48%
	Dhakal et al. (2011) [18]	SBA usage	31%
	Mayhew et al. (2008) [20]	SBA usage	13%
	Kempe et al. (2013) [31]	TBA usage (untrained)	71 (32%)
		TBA usage (trained)	10 (5%)
		No one attended delivery	30 (14%)
		Attended by medical doctor	37 (17%)
		Attended by nurse-midwife	29 (13%)
Facility-based delivery (FBD)	Devkota and Bhatta (2011) [17]	Facility-based delivery	8.5%
	Giacaman et al. (2006) [33]	Facility-based delivery	96.5%
		Delivered in govt hospital	56.4%
		Delivered in private hospital	28.3%
	Karkee et al. (2013) [19]	Facility-based delivery	85%
Postnatal care (PNC)	Basaleem (2012) [30]	Received any PNC	20%
	Dhaher et al. (2008) [32]	Received any PNC	36.6%
	Dhakal et al. (2011) [18]	Received any PNC	34%
		Received any within 48 h of birth	19%
		Received from a hospital	78%
		Received from a trained physician	65%
		Received from a nurse	20%
		Received from another health-worker	16%
Newborn care	Devkota and Bhatta (2011) [17]	Breastfed within 1 h of delivery	7%
	Najem and Al-Deen (2011) [28]	Breastfed within 1 h of delivery	7%
		Never breastfed	13.5%
	Ulak et al. (2012) [23]	Breastfed within 1 h of delivery	57%
	Devkota and Bhatta (2011) [17]	Did not seek health services for newborn complications	70.4%
		Did not vaccinate newborn	35.2%

Five studies (27%) mentioned SBA usage [16–18, 20, 31]. There was a sizable difference in SBA usage across studies from 13% in Nepal to 50% in Yemen [20, 30]. Kempe et al. [31] noted usage of untrained traditional birth attendants (32%) was much higher than of medical doctors (17%) or nurse-midwives (13%) in Yemen. Three studies discussed institutional delivery [17, 19, 33], which ranged from 85% in Nepal to 97% in Palestine [19, 33]. Giacaman et al. [33] noted that usage was higher for government (56%) compared to private (28%) facilities for childbirth in the Palestinian Territories.

Three studies (17%) discussed PNC, with less than 40% coverage found in Palestine, Yemen and Nepal [18, 30, 32]. More than 70 and 60% of those sampled received PNC from hospitals and trained physicians respectively [18]. Two studies (11%), from Nepal and Iraq, discussed newborn care [17, 23, 28]. Breastfeeding within an hour of delivery ranged from 7 to 57%. In Nepal and Iraq studies, 70% of mothers did not seek any professional advice on newborn health and 30% did not vaccinate newborns [16, 28].

### Determinants of service usage

**Fig. 2:**
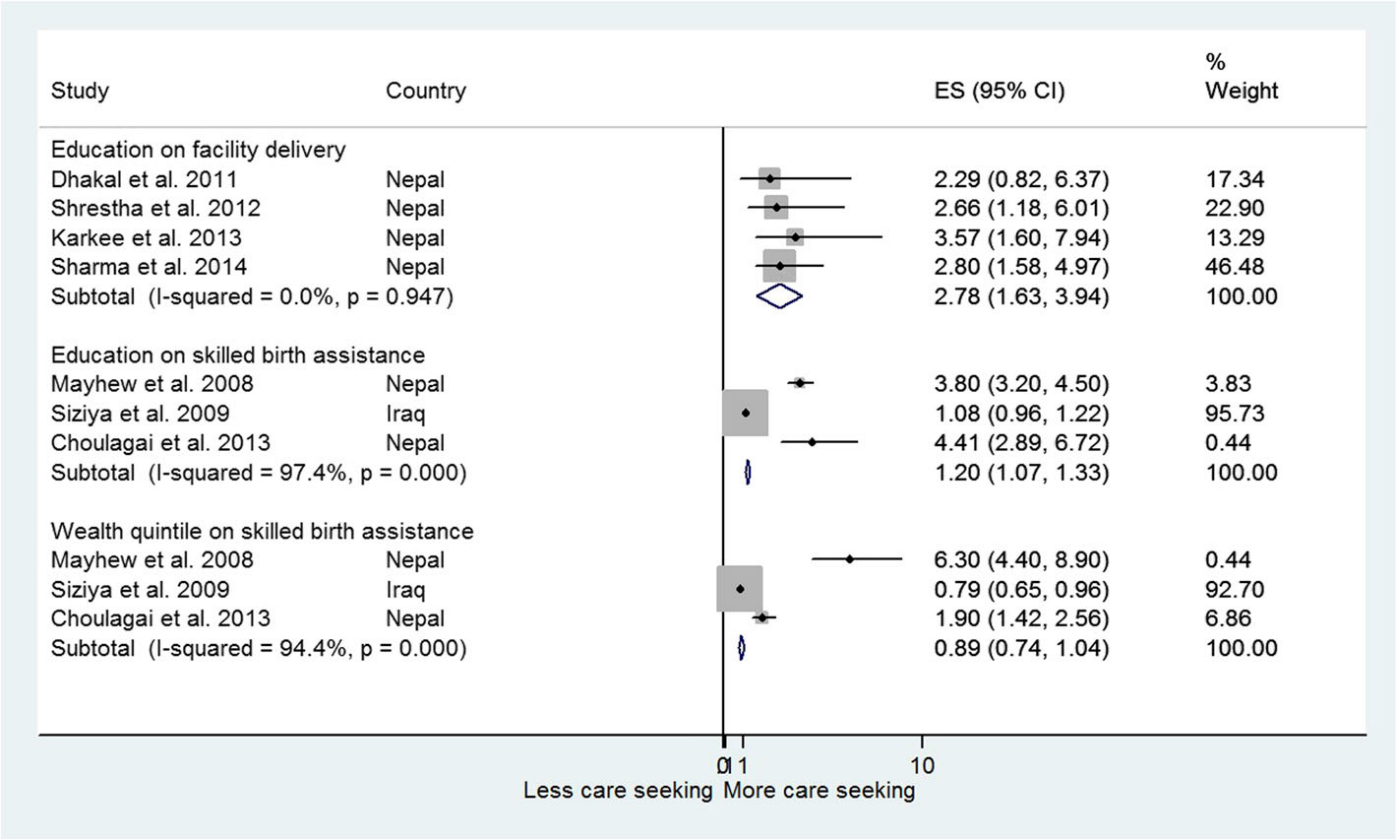
Association of determinants with MNH care seeking. NB: ES is the estimated statistic (odds ratio); weight, assigned weights by study in the estimation of pooled estimate

Table 5 summarises findings on determinants of service usage. Determinants of SBA were discussed in three studies from Iraq and Nepal [16, 20, 29]. Women who depended on TBAs for delivery were generally less educated, less wealthy, and older [29]. Having at least four ANC visits, knowledge of pregnancy danger signs, residing near a facility, being wealthier and better-educated were demand-side factors increasing the probability of SBA usage [16, 20, 29]. Pooled odds ratios (Fig. 2) showed education was associated with a 20% increased odds of SBA usage (OR 1.2, 95% CI 1.07-1.33), whereas wealth quintile was not significant (OR 0.89, 95% CI 0.74-1.04). On the supply-side, having more than one female traditional birth attendant (TBA) and community health-worker in the community, and at least one female doctor and provision of antenatal and emergency obstetric care in the facility increased SBA usage [20]. Similarly, absence of user fees and availability of health insurance increased the odds of SBA usage [20]. Qualitative studies indicated that women’s autonomy over decision-making about her health and personal life was a decisive factor in using SBA [31]. However, despite having autonomy several nomadic women in Yemen reported not wanting SBA [26, 31]. Two studies (11%) indicated that informal payments restricted timely access to facility care among pregnant women [26, 31].

**Table 5:** Factors affecting maternal and neonatal service usage, ordered by service type and outcome

Author (year), country	Outcomes	Determinants	Odds ratio or percentage
Skilled birth attendance			
Choulagai et al. (2013) [16], Nepal	SBA usage	Education	
		Informally educated Educated intermediate and above	OR 1.18 (CI0.92–1.51), *p* < 0.05 OR 4.41 (CI2.89–6.72), *p* < 0.05
		Wealth quintile	
		Q2 (poorer) Q5 (wealthiest)	OR 1.08 (CI 0.81–1.43), *p* < 0.05 OR 1.90 (CI 1.42–2.56), *p* < 0.05
		Knowledge	
		Knowledge of at least one danger sign	OR 1.31 (CI 1.08–1.58), *p* < 0.05
		Distance	
		Staying ≤ 30 min from facility	OR 1.31 (CI 1.08–1.58), *p* < 0.05
		ANC use	
		≥ 4 ANC visits	OR 2.39 (CI 1.97–2.89), *p* < 0.05
Mayhew et al. (2008) [20], Nepal	SBA usage	Wealth quintile	
		Q2 (poorer) Q5 (wealthiest)	OR 1.6 (CI 1.2–2.3), *p* < 0.01 OR 6.3 (CI 4.4–8.9), *p* < 0.01
		Distance	
		Walking distance to clinic (31–60 min) Walking distance to clinic (>90 min)	OR 0.7 (CI 0.6–0.8), *p* < 0.01 OR 0.4 (CI 0.3–0.6), *p* < 0.01
		Education	
		Formally educated	OR 3.8 (CI 3.2–4.5), *P* < 0.05
		Earlier been to this health facility	OR 1.7 (CI 1.3–2.1), *p* < 0.05
		At least some basic EmONC equipment in facility	OR 1.0 (CI 0.7–1.3), *p* < 0.05
		≥ 1 Community health worker in catchment area	OR 0.7 (CI 0.6–0.95), *p* < 0.05
		≥ 1 female TBA in catchment area	OR 1.3 (CI 1.0–1.7), *p* < 0.05
		≥ 1 female doctor or midwife at health facility	OR 1.4 (CI 1.1–1.8), *p* < 0.05
		User fees collected in facility	OR 0.8 (CI 0.6–0.96), *p* < 0.05
		Antenatal care provided in facility	OR 1.1 (CI 0.8–1.5), *p* < 0.05
Siziya et al. (2009) [29], Iraq	TBA usage	Wealth quintile	
		Q2 (poorer) Q4 (wealthier)	OR 2.90 (CI 2.49–3.39), *p* < 0.05 OR 0.79 (CI 0.65–0.96), *p* < 0.05
		Age	
		Women aged 25–34 years	AOR 1.22 (CI 1.08–1.39), *p* < 0.05
		Education	
		Formally educated	OR 1.08 (CI 0.96–1.22), *p* < 0.05
		Children	
		Having 1–2 children	AOR 0.72 (CI 0.59–0.87), *p* < 0.05
Facility-based delivery			
Dhakal et al. (2011) [18], Nepal	FBD	Age	
		25+ years 20–24 years	OR1.38 (CI 0.34–5.55), *p* < 0.001 OR 2.67 (CI 0.70–10.19), *p* < 0.001
		Occupation	
		Housewife Working women	OR 4.77 (CI 2.16–10.54), *p* < 0.001 OR 5.80 (CI 0.91–36.84), *p* < 0.001
		Education	
		Educated up to primary level Educated to secondary and above	OR 2.29 (CI 0.82–6.37), *p* < 0.001 OR 16.59 (CI 6.27–43.80), *p* < 0.001
		ANC use	
		≥ 1 ANC visit	OR 20.0 (CI 2.64–151.51), *p* < 0.001
Giacaman et al. (2006) [33], Palestine	FBD	Client satisfaction	
		Avoiding public facilities due to dissatisfaction	OR 2.77 (CI 1.89–4.05), *p* < 0.001
		Financial reasons	
		Insurance or low cost for opting facility	OR 5.83 (CI 3.96–8.59), *p* < 0.001
Karkee et al. (2013) [19], Nepal	FBD	Education	
		Educated up to primary level Educated to higher-secondary or above	AOR 3.57 (CI 1.60–7.94), *p* < 0.001 AOR 12.39 (CI 5.09–30.2), *p* < 0.001
		ANC use	
		≥ 4 ANC visits	AOR 2.15 (CI 1.25–3.69), *p* < 0.005
		Distance	
		≤ 30 min 31–60 min	OR 11.61 (CI 5.77–24.0), *p* < 0.001 AOR 1.72 (CI 0.93–3.19), *p* < 0.001
Sharma et al. (2014) [21], Nepal	FBD	Education	
		Formally educated	OR 2.8 (CI 1.58–4.97), *p* < 0.001
		Distance	
		< 60 min to facility	OR 3.12 (CI 1.61–0.04), *p* < 0.001
		ANC use	
		Had antenatal visits	OR 5.82 (CI 2.95–11.5), *p* < 0.001
Shrestha et al. (2012) [22], Nepal	FBD	Distance	
		Residing in remote area	OR 2.81 (CI1.08–7.30), *p* < 0.05
		Community	
		Newer community	OR 2.56 (CI 1.19–5.55), *p* < 0.05
		Education	
		Formally educated	OR 2.66 (CI 1.18–6.01), *p* < 0.05
		ANC use	
		No ANC visits	OR 5.53 (CI 2.12–14.4), *p* < 0.05
Emergency obstetric care			
Hirose et al. (2011) [24], Afghanistan	Delay in seeking EmONC	ANC use	
		Lack of ANC	AOR 4.6 (CI 1.7–12.2), *p* < 0.05
		Socio-cultural factors	
		Usage of traditional healer Weak relationship with her birth family No plan to use health facility for delivery	AOR 3.2 (CI 1.2–8.5), *p* < 0.05 AOR 2.0 (CI 0.9–4.4), *p* < 0.05 AOR 2.0 ( CI 0.9–4.2), *P* < 0.05
		System factors	
		Absence of a midwife	AOR 2.2 (CI 1.1–4.5), *p* < 0.05
Khorrami et al. (2008) [25], Afghanistan	Timely EmONC usage	Distance to facility	
		< 100 miles ≥ 100 miles	*N* = 249 (85.3%) *N* = 43 (14.7%)
		Mode of travel	
		Automobile Bus	*N* = 192 (65.8%) *N* = 97 (33.2%)
		Cost as a limitation	
		Yes No	*N* = 38 (30.7%) *N* = 86 (69.4%)
		Safety felt about care at this hospital	
		Moderately safe Mildly safe	*N* = 100 (34.4%) *N* = 177 (60.8%)

NB: CI is 95% confidence interval. *AOR* is adjusted odds ratio

Determinants of facility-based delivery were discussed in five (27%) studies [19, 21, 22, 29, 33]. Usage was higher among women with at least one ANC visit, more education, younger, employed, belonging to higher-status social groups, and who resided near facilities and in physically-accessible areas [18, 19, 21, 22, 33]. Meta-analysis indicated education was a significant determinant across most studies. Pooled odds ratios showed education was significantly associated with a higher proportion of facility-based delivery (OR 2.78, 95% CI 1.63-3.94). Qualitative research in Yemen indicated women preferred facility-based delivery given adequate staff, medicines, emergency care, cleanliness and arrangements for labour induction and neonatal resuscitation [30]. Public hospitals were reportedly preferred by Yemeni women due to the presence of qualified staff. [30] However, some women preferred home delivery for feelings of security, family support, and lower costs [30].

Determinants of delayed EmONC was discussed in two (11%) studies [24, 25]. These included not attending any ANC, care-seeking from traditional healers, and not wanting facility-based delivery. Absence of a midwife in the community or a facility also contributed to delayed EmONC. One study showed that residing near a facility, availability of an automobile for travel, inexpensive services, and perceived availability of safe care at the facility all increased the odds of EmONC usage [25].

## Discussion

### Summary of evidence

This systematic review synthesized the limited qualitative and quantitative evidence on maternal and neonatal service usage and its determinants in fragile and conflict-affected situations in Asia and the Middle-East. Relevant findings are similar to those in non-FCS, but more difficult to resolve given the unique challenges of FCS. These include disparities in the usage of essential MNH services among different socioeconomic, demographic and geographic groups even within FCS, with disadvantaged groups (e.g. women with no formal education, living at a distance from health facilities) facing additional barriers. This highlights the need to improve delivery of key MNH services (e.g. SBA, EmONC, PNC) in Asian and Middle-Eastern FCS. Similarly, recognised demand-side barriers from non-FCS, including lack of awareness, money, and transportation, still need to be sufficiently addressed in Asian and Middle-Eastern FCS. This review revealed evidence gaps in MNH in Asian and Middle-Eastern FCS, particularly for EmONC, PNC, newborn care, and service usage in acute-conflict situations.

Both low- and middle-income countries were included in this review, and usage of MNH services and their determinants were found to be similar across both categories. These findings reiterate the similarly limited healthcare infrastructure, service delivery and demand-side pre-disposing factors, found in FCS irrespective of national income [34, 35]. Most studies focused on remote and marginalised populations within FCS, which while useful in describing in-country social and political fragility, limited generalisability. No studies distilled MNH service-usage during acute conflict, though Afghanistan, Iraq and the Palestinian Territories included areas of acute conflict. Existing evidence indicates that even better-performing FCS are unable to effectively address MNH during recurrent crises [6, 36, 37]. Without ensuring EmONC and transportation services during acute crises, maternal and neonatal deaths in FCS will not be curtailed [6]. Additional research is needed to explore MNH services access and adaptive responses during acute crises (e.g. EmONC) to improve services sufficiently to achieve global MNH targets [36].

Evidence is particularly limited on usage of some essential maternal and neonatal health services for EmONC, PNC, and newborn care in the FCS of Asia and Middle-East. Overall, MNH service usage trends in Asian and Middle-Eastern FCS were similar to those found for FCS globally (e.g. low SBA and PNC usage) [38–43]. Usage of all MNH services was low, though highest for ANC. However, data collection emphasised receiving any ANC rather than the 4+ ANC visits recommended by WHO. Thus, it is not possible to conclude that existing ANC usage is adequate in these FCS. More research is necessary on timing of first visit and completion of 4+ ANC visit [7, 20].

SBA usage differed significantly across studies within the same countries, though usage was universally low. This intra-country variation appears due to socioeconomic differences, with SBA usage higher in relatively wealthier areas within countries (e.g. Nepal). Additionally, some differences could be attributed to substantial variations on sample sizes across studies. In FCS, presence of a female traditional birth attendant (TBA) or community health worker (CHW) increased the possibility of receiving SBA. As demonstrated by several other resource-constraint settings (e.g. Kenya and Uganda), mainstreaming TBA or CHW into formal primary care system can be a feasible option to improving access to MNH care in FCS as well [44, 45].

Facility-based delivery was higher than SBA usage, and highest in the Palestinian Territories. Literature indicates that the Palestine Territories, despite fragility and ongoing conflict has a comparatively higher rate of dependence on skilled professionals for maternal care, even in refugee camps, though quality of care is a concern [34, 35]. Small geographical size of the territories likely contributes to higher SBA usage [37]. Elsewhere, dependence on TBAs was considerable, and generally attributed to inadequate availability of professional care and potential lack of awareness [31]. Over-reliance on TBAs is commonly reported in FCS globally due to demand- and supply-side barriers to usage of professional services [36, 37].

Usage of newborn services was mixed. Usage of public facilities was higher than for private facilities in these FCS [30], a trend recognised in FCS globally (e.g. Zimbabwe, Somalia) [7, 38, 39]. While findings were mixed on initiation of early breastfeeding within an hour of birth, no study found timely care-seeking for neonatal health concerns. Several women reported using traditional healers instead, as reported in other FCS [7, 38–43].

It is essential to highlight the fundamental inequity found in MNH services usage in the studies included. Both demand- and supply-side factors favoured wealthier women. For example, MNH services usage was higher among women who were wealthier and in geographically-accessible areas. It is likely these women had more knowledge, income, and/or available services encouraging greater usage. Inequity in MNH services access is found in both FCS and non-FCS (e.g. Nigeria, Uganda) [41, 42] as social programmes tend to favour advantaged populations through existing structures and practices in coverage and implementation [42]. However, FCS must address a relatively stronger vicious circle of inequity due to weak governance and policy paralysis, which augment these inequities [46, 47].

To address the barriers and inequities discussed, FCS require more support than do non-FCS. However, international donor presence does not necessarily streamline health policy-making in FCS [2, 3]. For example, though targeted policies and strategies for vulnerable groups were planned by donors, often no commensurate improvement in living and health standards is visible, due to issues such as weak aid coordination, planning and institutional support. [2–4] Authors propose adapting targeted policy approaches to improve coordination of aid and planning. Specific demand-side initiatives are needed to address socio-cultural barriers and women’s financial access to healthcare in FCS. Given the often limited finances available to women in FCS, policy-level action is necessary to reduce demands for informal payments [26, 31]. Supply-side issues with availability of community-based workers and transportation services were emerging themes that require additional research.

### Limitations

This review is limited by the studies available. Though incorporating a vast literature, this review included only peer-reviewed research articles with a minimum level of methodological quality, thus considerable gaps remain. Authors adhered to the FCS classification used by the World Bank, which may have omitted some potential fragile countries in Asia or the Middle-East. Additionally, authors did not analyse sub-national fragility and therefore omitted non-FCS with higher burdens of maternal and neonatal ill-health (e.g. India) [47]. Caution should be used in interpreting findings, as studies included were primarily non-generalisable.

Despite limitations, this review is relevant for contributing analysis of the evidence on MNH service-usage in FCS and potentially increasing policy attention for this under-researched topic. As this review covers the relatively under-studied factors associated with MNH and service-usage in Asian FCS, inclusion of both qualitative and quantitative data strengthened potential triangulation.

## Conclusions

Findings emphasise that poor MNH in FCS is a leading contributor to the burden of maternal and neonatal ill-health in Asia and Middle-East. FCS require additional resources and policy attention if they are to address key barriers to effective MNH care. While existing evidence is relatively minimal, particularly for acute-crises, similarities in MNH service-usage and determinants were found in Asian and Middle-Eastern FCS. Overall, usage remains low due to supply-side (e.g. limited services, quality, costs) and demand-side (e.g. education) barriers. More evidence is necessary on timing of first ANC visit, completion of 4+ ANC visits, and increasing usage of EmONC, PNC and newborn services. Initiatives to increase effective coverage of SBA, EmONC, PNC and newborn services are particularly needed, while prevailing service-usage inequities can be tackled through targeted policy approaches.

## Abbreviations

ANC: Antenatal care
EmONC: Emergency obstetric and newborn care
FCS: Fragile and conflict-affected situations
MNH: Maternal and neonatal health
PNC: Postnatal care
SBA: Skilled birth attendance
